# Molecular Detection and Characterization of *Mycoplasma* spp. in Marine Mammals, Brazil

**DOI:** 10.3201/eid2912.230903

**Published:** 2023-12

**Authors:** Aricia Duarte-Benvenuto, Carlos Sacristán, Ana Carolina Ewbank, Roberta Zamana-Ramblas, Henrique Christino Lial, Samira Costa Silva, Maria Alejandra Arias Lugo, Lara B. Keid, Caroline F. Pessi, José Rubens Sabbadini, Vanessa L. Ribeiro, Rodrigo del Rio do Valle, Carolina Pacheco Bertozzi, Adriana Castaldo Colosio, Hernani da Cunha Gomes Ramos, Angélica María Sánchez-Sarmiento, Raquel Beneton Ferioli, Larissa Pavanelli, Joana Midori Penalva Ikeda, Vitor L. Carvalho, Felipe Alexandre Catardo Gonçalves, Pablo Ibáñez-Porras, Irene Sacristán, José Luiz Catão-Dias

**Affiliations:** University of São Paulo, São Paulo, Brazil (A. Duarte-Benvenuto, A.C. Ewbank, R. Zamana-Ramblas, H.C. Lial, S.C. Silva, M.A.A. Lugo, L.B. Keid, J.L. Catão-Dias);; Centro de Investigación en Sanidad Animal, Madrid, Spain (C. Sacristán, P. Ibáñez-Porras, I. Sacristán);; Instituto de Pesquisas Cananéia, Cananéia, Brazil (C.F. Pessi, J.R. Sabbadini);; Instituto Biopesca, Praia Grande, Brazil (V.L. Ribeiro, C.P. Bertozzi);; Aiuká Consultoria Ambiental, Praia Grande (R. del Rio do Valle);; Universidade Estadual Paulista, Campus do Litoral Paulista, São Vicente, Brazil (C.P. Bertozzi);; Instituto Baleia Jubarte, Caravelas, Brazil (A. Castaldo Colosio, H. da Cunha Gomes Ramos);; Instituto Argonauta para a Conservação Costeira e Marinha, Ubatuba, Brazil (A.M. Sánchez-Sarmiento, R. Beneton Ferioli);; Instituto Mamíferos Aquáticos, Salvador, Brazil (L. Pavanelli, J.M. Penalva Ikeda);; Associação de Pesquisa e Preservação de Ecossistemas Aquáticos, Caucaia, Brazil (V.L. Carvalho, F.A. Catardo Gonçalves)

**Keywords:** Mycoplasma, hemoplasmas, hemotropic Mycoplasma, bacteria, zoonoses, cetaceans, pinnipeds, Brazil, South America

## Abstract

*Mycoplasma* spp. are wall-less bacteria able to infect mammals and are classified as hemotropic (hemoplasma) and nonhemotropic. In aquatic mammals, hemoplasma have been reported in California sea lions (*Zalophus californianus*) and river dolphins (*Inia* spp.). We investigated *Mycoplasma* spp. in blood samples of West Indian manatees (*Trichechus manatus*), pinnipeds (5 species), and marine cetaceans (18 species) that stranded or were undergoing rehabilitation in Brazil during 2002–2022. We detected *Mycoplasma* in blood of 18/130 (14.8%) cetaceans and 3/18 (16.6%) pinnipeds. All tested manatees were PCR-negative for *Mycoplasma*. Our findings indicate that >2 different hemoplasma species are circulating in cetaceans. The sequences from pinnipeds were similar to previously described sequences. We also detected a nonhemotropic *Mycoplasma* in 2 Franciscana dolphins (*Pontoporia blainvillei*) that might be associated with microscopic lesions. Because certain hemoplasmas can cause disease and death in immunosuppressed mammals, the bacteria could have conservation implications for already endangered aquatic mammals.

Marine mammals are a diverse polyphyletic group of animals that are anatomically and physiologically adapted to an aquatic lifestyle and depend on marine ecosystems (*1*). The group includes members of the order Carnivora, such as pinnipeds, sea otters (*Enhydra lutris*), marine otters (*Lontra felina*), and polar bears (*Ursus maritimus*); order Sirenia, which includes manatees and dugongs (*Dugong dugon*); and the infraorder Cetacea, which comprises dolphins, whales, and porpoises (*1*,*2*). Those diverse species are considered ecosystem sentinels (*3*,*4*). The coast of Brazil sustains a high diversity of marine mammals, including 44 cetacean, 8 pinniped, and 1 sirenian species (*5*,*6*), at least 8 of which are classified into some level of extinction risk (*7*,*8*). Anthropogenic threats, such as pollution, climate change, and bycatch, severely effect marine mammal populations; however, infectious diseases also play a role (*9*). Information on marine mammal diseases in Brazil is limited, particularly in sirenians, and usually focuses on viral infections (*10*–*14*).

Mycoplasmas are a group of pleomorphic cell wall–deficient bacteria of the class Mollicutes that are considered the smallest self-replicant prokaryotes (*15*). Mycoplasmas have been detected in several mammal species, including humans (*16**–**19*). *Mycoplasma* spp. are divided into 2 distinct groups on the basis of infection tropism: nonhemotropic mycoplasmas and hemotropic mycoplasmas (hemoplasmas) (*15*,*20*). Nonhemotropic mycoplasmas usually infect epithelial cells of the respiratory and urinary tracts and have been associated with persistent pneumonia, opportunistic nephritis, encephalopathies, autoimmune disease, and reproductive disorder in multiple species (*21*–*23*). Hemotropic mycoplasmas can adhere to erythrocyte surfaces and have been linked to acute and chronic anemia, starvation, and even death, especially when they occur along with other pathogens or in immunosuppressed or immature mammals (*24*). Regardless, most hemoplasma species cause subclinical infections (*19*,*24*).

Available studies have focused on domestic animals, but hemoplasmas have attracted the attention of wildlife researchers in the past decade (*23*). Hemoplasmas have high infection prevalence and genetic diversity, and hemoplasma DNA has been found in several wild mammals (*25*,*26*). In addition, occasional reports of interspecies transmission could indicate zoonotic potential (*17*,*27*). Despite the potential for zoonoses, few taxa have been described, and little is known about hemoplasma transmission routes and pathogenesis (*19*).

Among marine mammals, hemoplasmas have only been reported in pinnipeds, and a study of California sea lions (*Zalophus californianus*) described an infection rate of 12.4% (17/137) (*28*). We recently detected hemoplasma in 65.6% (32/50) of sampled river dolphins (*Inia geoffrensis* and *I. boliviensis*) from the Amazon Basin, indicating that the bacteria are also circulating in fully aquatic mammals (*13*). Nonhemotropic mycoplasmas have been detected in marine mammals, particularly in pinnipeds, but only a few cases have been reported in cetaceans (*29*–*35*). Of note, *Mycoplasma phocicerebrale*, one of the species isolated from harbor seals (*Phoca vitulina*) in 1988, is recognized as the likely cause of seal finger disease in humans, which is transmitted by direct contact with infected seals through bites or handling (*35*). Despite those reports, little information is available on hemotropic mycoplasma in marine cetaceans and sirenians worldwide or on epitheliotropic mycoplasma in cetaceans of the Southern Hemisphere. We surveyed and characterized *Mycoplasma* spp. in blood samples of marine mammals that were stranded or undergoing rehabilitation in Brazil during 2002–2022.

## Materials and Methods

### Samples

All collected samples described herein are stored in the Marine Mammal Blood and Tissue Bank of the Laboratory of Wildlife Comparative Pathology of the University of São Paulo, São Paulo, Brazil. Samples are periodically surveyed for other pathogens depending on necropsy and histopathological reports from sampled mammals.

#### Cetaceans

We analyzed samples from 130 marine cetaceans that had stranded alive or dead or were bycaught (caught unintentionally) along the northeastern (62/130) or southeastern (68/130) coast of Brazil during 2011–2022. Samples were from animals of the families Delphinidae (n = 67), Pontoporiidae (n = 42), Kogiidae (n = 14), Balaenopteridae (n = 6), and Physeteridae (n = 1), comprising 18 different species (Table 1). Blood samples were collected from the ventral caudal peduncle in live animals or directly from the heart in dead stranded animals, placed in vacutainer tubes with EDTA, and maintained at −80°C until analysis.

**Table 1:** Taxonomic and sex information on cetaceans in a study of molecular detection and characterization of *Mycoplasma* spp. in marine mammals, Brazil*

Cetacean scientific name	Common name	Sex		Total	No. (%) *Mycoplasma*-positive
		F	M		
Balaenopteridae					
* Megaptera novaeangliae*†	Humpback whale	0	5	6	0
Delphinidae					
* Delphinus delphis*	Common dolphin	1	1	2	1 (50)
* Feresa attenuata*	Pigmy killer whale	1	3	4	1 (25)
* Globicephala macrorhynchus*	Short-finned pilot whale	2	0	2	0
* Grampus griseus*	Risso’s dolphin	1	0	1	0
* Lagenodelphis hosei*	Fraser’s dolphin	4	1	5	0
* Orcinus orca*	Killer whale	1	0	1	1 (100)
* Peponocephala electra*	Melon headed whale	1	9	10	0
* Sotalia guianensis*	Guiana dolphin	13	11	24	3 (12.5)
* Stenella clymene*	Clymene dolphin	1	2	3	0
* Stenella coeruleoalba*	Striped dolphin	2	0	2	1 (50)
* Stenella frontalis*	Atlantic spotted dolphin	4	3	7	2 (28.5)
* Stenella longirostris*	Spinner dolphin	0	2	2	0
* Steno bredanensis*	Rough-toothed dolphin	1	3	4	1 (25)
Kogiidae					
* Kogia breviceps*	Pigmy sperm whale	1	3	4	3 (75)
* Kogia sima*	Dwarf sperm whale	4	6	10	2 (20)
Physeteridae					
* Physeter macrocephalus*	Sperm whale	1	0	1	0
Pontoporiidae					
* Pontoporia blainvillei*	Franciscana dolphin	21	21	42	3 (7.1)
Total		59	70	130	18

*Mammals were stranded or caught along the coast of Brazil during 2011–2022. †One animal’s sex was not determined.

Animals that stranded dead or that died during rehabilitation were necropsied according to standard procedures (*36*). Age class was established according to total body length (*6*). Selected tissue samples were fixed in 10% formalin at room temperature. An additional set of samples, including lung, spleen, cerebrum, and liver, was frozen at −80°C until analysis.

#### Pinnipeds

We analyzed blood samples from 18 pinnipeds that stranded alive during 2018–2022 along the northeastern (1/18) or southeastern (17/18) coast of Brazil, including 9 South American fur seals (*Arctocephalus australis*), 4 subantarctic fur seals (*A. tropicalis*), 2 Antarctic fur seals (*A. gazella*), 1 crabeater seal (*Lobodon carcinophaga*), and 1 southern elephant seal (*Mirounga leonina*). Blood samples were collected from the caudal gluteal or interdigital veins, placed in vacutainer tubes with EDTA, and maintained at −80°C until analysis.

#### Sirenians

We analyzed samples of 24 West Indian manatees (*Trichechus manatus*) undergoing rehabilitation along the northeastern coast of Brazil. Blood samples were collected from the ventral pectoral fin, placed in vacutainer tubes with EDTA, and maintained at −80°C until processing. Of note, those blood samples were previously screened for herpesvirus and adenovirus (*14*).

### Ethics Approvals

This study was approved by the Ethical Committee in Animal of the School of Veterinary Medicine and Animal Sciences, University of São Paulo (process no. 1698290119). The collection and transportation of all samples was approved by the Brazilian Institute of the Environment and Natural Renewable Resources (license no. 67766). The genetic analysis was approved by the National System for the Management of Genetic Heritage and Associated Traditional Knowledge (approval no. A49250C).

### Molecular Assays

We extracted total DNA from 172 blood samples by using the DNeasy Blood & Tissue Kit (QIAGEN, https://www.qiagen.com), according to the manufacturer’s instructions. We screened samples for *Mycoplasma* spp. DNA by using a real time PCR protocol targeting a 384-bp fragment of the 16S rRNA gene that we adapted from a previous study (*25*). To molecularly characterize the bacteria, we further subjected confirmed positive samples to a nested PCR targeting a 1,100–1,400-bp fragment of the 16S rRNA gene (*37*) and to a conventional PCR targeting an 800-bp fragment of the 23S rRNA gene (*38*). In addition, we extracted and tested 8 tissue samples collected from lung, spleen, cerebrum, and liver of a Franciscana dolphin (*Pontoporia blainvillei*, Pontoporiidae family) that tested positive for nonhemotropic mycoplasma in blood and of its calf, which was found dead alongside the female, to further investigate a potential systemic infection.

We purified positive amplicons by using ExoSAP-IT PCR Product Cleanup (Affymetrix–Thermo Fisher Scientific, https://www.thermofisher.com) and GFX PCR DNA and Gel purification (Global Life Sciences Sigma-Aldrich, https://www.sigmaaldrich.com), and confirmed by direct Sanger sequencing in both directions. We assembled sequence reads in MEGA 7.0 (*39*) by using ClustalW (http://www.clustal.org/clustal2) alignment and compared sequences with those available in GenBank by searching BLASTn (https://blast.ncbi.nlm.nih.gov). We calculated nucleotide and amino acid genetic distances to the closest sequences on the basis of p-distance, after editing out the primers. Finally, we used MEGA 7.0 to construct nucleotide maximum-likelihood phylogenetic trees with a bootstrap value of 1,000 replicants and a general time-reversible plus invariant site model for 23S gene and Tamura 3 parameter with inversions and gamma distribution model for the 16S gene (329 bp). We selected those evolutionary models by using jMODELTEST 2.1.10 (My Biosoftware, https://mybiosoftware.com). We omitted all bootstrap frequency values <70. 

### Statistical Analysis

We performed statistical analyses in GraphPad Prism version 5 (GraphPad Software Inc., https://www.graphpad.com) to establish whether hemoplasma could be associated with the any of the following variables: host family, habitat distribution, sampling region, sampling year, age, or sex. We considered p<0.05 statistically significant. We used χ^2^ test to analyze differences between sexes and Kruskal–Wallis test to analyze the remaining variables. None of the analyzed variables were statistically significant.

### Histopathologic Examination

We analyzed available formalin-fixed samples from 2 positive cases of nonhemotropic *Mycoplasma*, a Franciscana dolphin (identification [ID] number 85) and its calf (ID173). We embedded samples in paraffin wax, processed according to routine procedures of the School of Veterinary Medicine and Animal Sciences of University of São Paulo, sectioned at 5 μm, and stained with hematoxylin and eosin for light microscopic examination.

## Results

### Molecular Findings

#### Cetaceans

**Figure 1:**
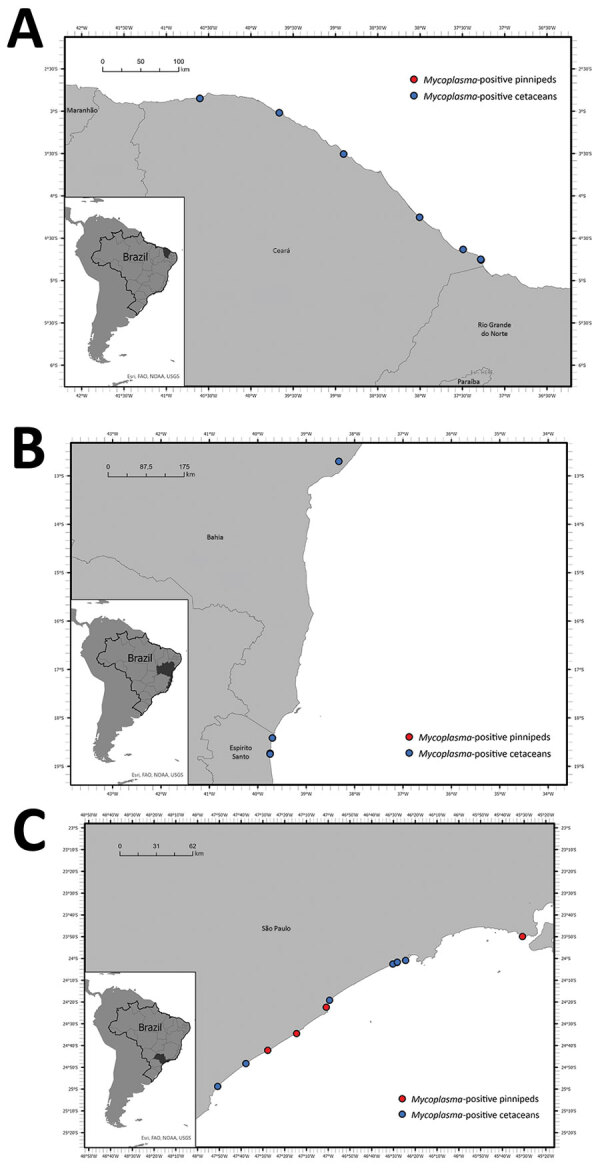
Stranding locations of *Mycoplasma*-positive cetaceans and pinnipeds sampled for a study of molecular detection and characterization of *Mycoplasma* spp. in marine mammals, Brazil. A) Ceará state, in the northeast region; B) Bahia and Espírito Santo states; C) São Paulo state, in the southeast region. Insets show location of each region in Brazil. Dots indicate single animals that tested *Mycoplasma*-positive.

We detected *Mycoplasma* DNA in the blood of 13.8% (18/130) of tested animals by using the 16S rRNA gene real-time PCR (Figure 1), including 14.9% (10/67) of Delphinidae, 35.7% (5/14) of Kogiidae, and 7.1% (3/42) of Pontoporiidae (Table 1). None of the tested Balaenopteridae and Physeteridae were positive (Table 1). We compiled biologic and molecular data for the *Mycoplasma*-positive cases (Table 2).

**Table 2:** Biologic and molecular data from mammals in a study of molecular detection and characterization of *Mycoplasma* spp. in marine mammals, Brazil*

Case no.	Species	Age class/sex	Year stranded	16S rRNA sequence length, bp	Accession no.†	*Mycoplasma* sequence comparison		
						% nt Identity	Closest available sequence†	Species of detection
ID1	*Kogia breviceps*	Adult/M	2017	1,100	OR193701	98.9	ON721294	*Inia geoffrensis*
ID13	*Kogia sima*	Adult/F	2019	1,100	OR193699	98.9	ON721294	*I. geoffrensis*
ID14	*Stenella coeruleoalba*	Adult/F	2019	Inadequate sequence	NA	NA	NA	NA
ID15	*Kogia breviceps*	Adult/M	2020	1100	OR193701	98.9	ON721294	*I. geoffrensis*
ID16	*Kogia sima*	Adult/M	2020	1,100	OR193703	98.7	ON721294	*I. geoffrensis*
ID19	*Stenella frontalis*	Adult/F	2020	330	OR184994	92.3	OL985926	*Tapirus terrestris*
ID26	*Kogia breviceps*	Adult/F	2020	1,100	OR193701	98.7	ON721294	*I. geoffrensis*
ID29	*Stenella frontalis*	Adult/F	2021	Inadequate sequence	NA	NA	NA	NA
ID34	*Feresa attenuata*	Adult/M	2015	1,100	OR193702	98.0	ON721301	*Inia boliviensis*
ID62	*Delphinus delphis*	Juvenile/M	2021	1,100	OR193698	98.4	ON721294	*I. geoffrensis*
ID63	*Sotalia guianensis*	Juvenile/F	2021	330	OR193744	98.6	ON721294	*I. geoffrensis*
ID79	*Pontoporia blainvillei*	Calf/M	2019	1,100	OR193700	98.5	ON721294	*I. geoffrensis*
ID81	*Steno bredanensis*	Adult/M	2019	1,100	OR193727	98.5	ON721294	*I. geoffrensis*
ID85	*Pontoporia blainvillei*	Adult/F	2018	1,100	OR183450	98.5	JN792314	*Neophocaena phocaenoides asiaeorientalis*
ID95	*Orcinus orca*	Juvenile/F	2020	1,100	OR193728	98.5	ON721294	*I. geoffrensis*
ID119	*Sotalia guianensis*	Juvenile/M	2022	330	OR193742	100	ON721299	*I. geoffrensis*
ID122	*Pontoporia blainvillei*	Calf/F	2022	330	OR193741	99.7	ON721301	*I. boliviensis*
ID126	*Sotalia guianensis*	Juvenile/M	2022	330	OR193743	98.6	ON721301	*I. boliviensis*
ID131	*Arctocephalus tropicalis*	Adult/M	2020	1,100	OR193739	100	GU124613	*Zalophus californianus*
ID132	*Arctocephalus tropicalis*	Adult/F	2020	1,100	OR193740	99.3	GU124613	*Z. californianus*
ID144	*Arctocephalus gazella*	Juvenile/M	2021	410	OR193745	99.3	GU124613	*Z. californianus*

*Mammals were stranded or caught along the coast of Brazil during 2011–2022. bp, base pairs; ID, identification; NA, not applicable. †From GenBank.

After sequencing the 16S rRNA fragments, we were able to retrieve 1,100-bp sequences from 11 cases and 330-bp sequences from another 5 cases, comprising a total of 12 sequence types. In addition, we confirmed *Mycoplasma* in 2/18 sequences; however, the low sequence quality prevented species characterization. Among the good quality sequences, 14 *Mycoplasma* nucleotide sequences from marine dolphins in our study showed >98.0% nt identity with the closest available sequences (GenBank accession nos. ON721294, ON721301, and ON721299), which were previously detected in blood of riverine cetacean species (*15*). *Mycoplasma* sequences in our study were from Guiana dolphin (*Sotalia guianensis*), pygmy sperm whale (*Kogia breviceps*), dwarf sperm whale (*Kogia sima*), pygmy killer whale (*Feresa attenuata*), common dolphin (*Delphinus delphis*), Franciscana dolphin, killer whale (*Orcinus orca*), and rough-toothed dolphin (*Steno bredanensis*). All sequences retrieved from pygmy sperm whales were identical or very similar to sequences described in dwarf sperm whale and had only 2 single point mutations. We also detected a shared 1,100-bp sequence type in samples from a killer whale (ID95), a rough-toothed dolphin (ID81), and a Franciscana dolphin (ID79) that was very similar to the 1 detected in a common dolphin (ID62).

One of the 330-bp sequences detected in Atlantic spotted dolphin (*Stenella frontalis*) had 92.3% nt identity with an uncultured *Mycoplasma* spp. (GenBank accession no. OL985926) detected in lowland tapir (*Tapirus terrestris*) from Brazil (Table 2), likely representing a novel hemoplasma species. Of note, 1 of the retrieved consensus sequences from blood of a Franciscana dolphin had the highest nucleotide identity (98.5%) with a *Mycoplasma* spp. sequence identified in a fecal sample from Yangtze finless porpoise (*Neophocaena phocaenoides asiaeorientalis*) from China that was not classified as hemoplasma. In addition, we detected that nonhemotropic mycoplasma in spleen, liver, and lung of the same dolphin (ID85) and in the cerebrum, spleen, and lung of its calf (ID173).

**Figure 2:**
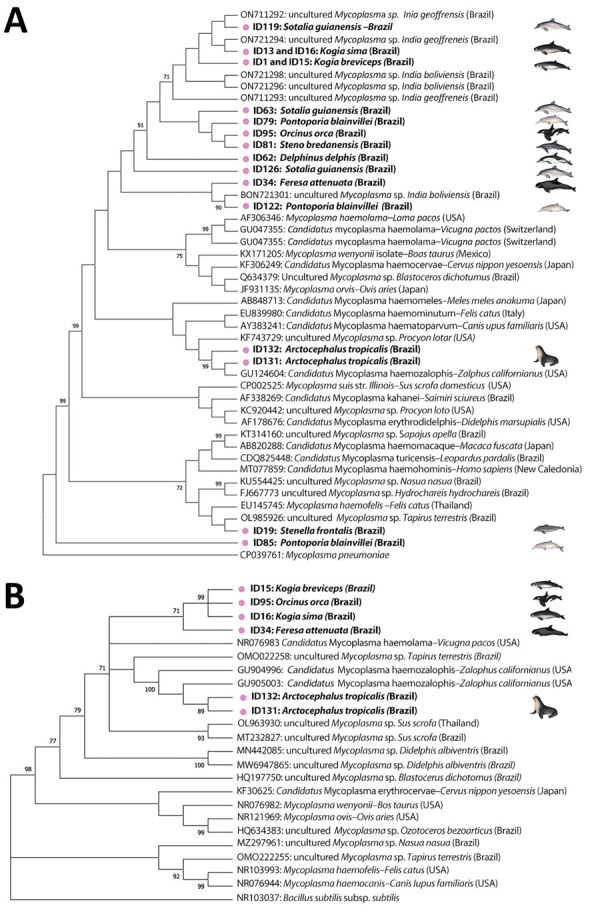
Maximum-likelihood phylograms from a study of molecular detection and characterization of *Mycoplasma* spp. in marine mammals, Brazil. A) 16S rRNA gene (329-bp) phylogram based on Tamura 3-parameter with inversions and gamma distribution model. *Mycoplasma pneumoniae* was selected as outgroup. B) 23S rRNA gene (820-bp) phylogram based on a general time-reversible model with invariant sites. *Bacillus subtilis* was selected as outgroup. Trees show alignment of *Mycoplasma* spp. consensus sequences obtained in marine mammals from this study (pink dots and bold text) and other hemotropic mycoplasmas retrieved from GenBank. Reliability of the phylograms was tested by 1,000 replicate bootstrap analyses omitting values <70. Trees generated by using MEGA 7.0 (https://www.megasoftware.net).

We were able to recover 23S rRNA *Mycoplasma* spp. genes from 4/18 16S rRNA real-time PCR–positive cases in a killer whale (1/1), a pygmy sperm whale (1/3), a dwarf sperm whale (1/2), and a pigmy killer whale (1/1) (Table 1). The retrieved 800-bp sequences confirmed 88.4%–90.9% nt identity with a sequence of *Candidatus* Mycoplasma haemolamae (GenBank accession no. CP003731) detected in alpacas (*Vicugna pacos*) from the United States (Appendix Figure 1). All but 2 sequences clustered in the 16S rRNA gene phylogram with other sequences from river dolphins within the *Candidatus* M. haemosuis group (Figure 2, panel A). The sequence retrieved from Atlantic spotted dolphin clustered with other sequences of hemotropic mycoplasma within the *M. haemofelis* group, and the sequence detected in Franciscana dolphin clustered with the epithelitropic mycoplasma *M. pneumoniae* (Figure 2, panel A). In the 23S rRNA phylogram, all cetacean species clustered together (Figure 2, panel B). 

#### Pinnipeds

We detected *Mycoplasma* DNA in 3 of 18 blood samples (Figure 1), comprising 2 subantarctic fur seals and 1 Antarctic fur seal. We were able to recover two 1,100-bp sequences and one 410-bp sequence of the 16S rRNA gene and two 800-bp sequences of the 23S rRNA gene (Table 1). Phylogenetic analysis of the 16S rRNA gene showed that the retrieved sequences had confirmed (99.3%–100% nt) identity with *Mycoplasma* previously detected in California sea lions (GenBank accession no. GU124613). Both retrieved sequences of the 23S rRNA gene confirmed (99.0% and 99.1% nt) identity with a hemotropic mycoplasma also detected in California sea lions (GenBank accession no. GU905003). On the phylogram, the retrieved sequences clustered with *Candidatus* M. haemozalophis within the haemosuis group in both genes (Figure 2). We submitted representative sequences to GenBank (accession nos. OR183450, OR184994, OR185401–4, OR193689, OR193690, OR193698–700, OR193703, OR193728, OR193739–45).

#### Sirenians

We analyzed samples of 24 West Indian manatees. All manatees tested PCR negative for *Mycoplasma* DNA.

### Histopathologic Findings

We summarized all epidemiologic and biologic data of the tested marine mammals (Appendix Table). We described anatomopathological findings of the Franciscana dolphin and calf (ID85 and ID173) that tested positive for nonhemotropic mycoplasma (Table 3; Appendix Figure 2). 

**Table 3:** Main pathologic findings of 2 bycaught Franciscana dolphins (*Pontoporia blainvillei*) positive for nonhemotropic mycoplasma in a study of molecular detection and characterization of *Mycoplasma* spp. in marine mammals, Brazil

Case no.	Gross findings	Microscopic findings
ID85	External examination: multifocal linear imprints and incisions associated with net entanglement. Multifocal moderate teeth marks in all ventral aspects, particularly in the pedunculus.	Spleen: moderate white pulp hyperplasia. Moderate diffuse congestion. Mild to moderate acute granulocytic splenitis. Mild to moderate expansion of the periarteriolar lymphoid sheaths with deposition of eosinophilic amorphous material and lymphocytolysis.
	Lungs: marked diffuse congestion and mild edema. Multifocal mild atelectasis areas	Lungs: moderate multifocal congestion and hemorrhage. Mild to moderate granulocytic pneumonia with interalveolar macrophages and pneumocytes. Mild multifocal atelectasis.
	Axillary and mesenteric lymph nodes: moderate lymphadenomegaly	Prescapular lymph node: mild to moderate acute granulocytic lymphadenitis with lymphocytolysis in germinal centers.
	Central nervous system: mild to moderate cerebral congestion.	Liver: mild ductal hyperplasia. Mild congestion.
		Thyroid, thymus, kidney, and pituitary and adrenal glands: Mild to moderate congestion.
		Tongue, cerebellum, brain, mesenteric lymph node, stomach, and intestines: no significant findings observed
ID173, calf of ID85	External examination: mild multifocal linear marks associated with net entanglement.	Central nervous system: Mild to moderate congestion. Mild satellitosis and gliosis. Mild neuronophagia.
	Lungs: marked congestion, mild to moderate pulmonary edema.	Lung: Marked diffuse congestion and hemorrhage associated with moderate to marked multifocal edema. Mild to moderate granulocytic pneumonia with exfoliation of macrophages and pneumocytes in alveoli. Rare megakaryocytes.
	Liver: Mild multifocal to coalescent pale areas. Moderate congestion.	Liver: mild microgoticular degeneration and congestion.
	Generalized congestion.	Kidney: mild congestion and hemorrhage.
		Spleen, skeletal muscle, adrenal glands, trachea, skin, stomach, mesenteric and rectal lymph nodes: no significant findings observed

*ID, identification.

## Discussion

We detected hemoplasma in cetaceans and pinnipeds in Brazil. We observed a 13.8% occurrence rate in tested cetaceans (18/130), which was lower than rates observed in river dolphins (64%; 32/50) but similar to the rates reported in California sea lions (12.4%; 17/137) (*13*,*28*). The spatial distribution of the marine cetaceans we studied is wider and less restricted than the previously studied river dolphin populations, which could affect hemoplasma circulation. Furthermore, the sampling method could have influenced the different occurrence rates among marine and riverine cetaceans. In this study, we sampled marine mammals involved in stranding events that were distributed over a broad geographic area and timeframe, and multiple species and groups were represented. Our previous study involved data collected in scientific captures concentrated in a few days and usually involving animals from the same family group and species (*13*).

Several previous hemoplasma studies targeted the 16S rRNA gene, a highly conserved genetic region, which could affect the identification of novel species because the gene can be undifferentiable between pathogen species (*19*). Despite targeting this gene, the nucleotide sequence we retrieved from an Atlantic spotted dolphin showed 92.3% nt identity with the closest sequence available, likely corresponding to a novel hemoplasma species. In the phylogram, that novel species did not cluster with the other hemoplasmas described in cetaceans to date, evidencing high divergency. The species also was classified within the *M. haemofelis* group (Figure 2), which usually is associated with higher pathogenicity. All the remaining hemoplasma sequences we retrieved from cetaceans had >98.0% nt identity with sequences of previously detected hemoplasma in river dolphins and clustered with those sequences. That high similarity among most of the obtained cetacean sequences likely indicates co-evolution or co-divergence of the pathogen and a common ancestry. Nevertheless, the novel sequence detected in an Atlantic spotted dolphin suggests that at least 2 different species of hemotropic mycoplasmas are circulating in cetaceans in the south Atlantic.

We detected the same 16S rRNA sequence type in 3 different species, Franciscana dolphin, killer whale, and rough-toothed dolphin, which belong to 2 different taxonomic families and the samples were collected in different regions of Brazil. That pattern diverged from the pattern we observed in river dolphins, where we could link genetic structure on the basis of host and collection site (*13*). That finding potentially indicates interspecies transmission of hemoplasma among marine cetaceans because those species are not geographically separated by natural barriers like the river dolphins we studied. We could only amplify the 23S gene in one of the mentioned animals, which precluded a multilocus analysis.

In pinnipeds, 16S and 23S sequences retrieved in subantarctic and Antarctic fur seals were very similar to sequences previously detected in California sea lions (>98.9% nt identity). Considering the geographic and taxonomic differences among those 3 host species, our findings could indicate a low mutation rate within this bacterium. Antarctic and subantarctic fur seals belong to the genus *Arctocephalus* and both have reproductive colonies near the Antarctic convergence in the Southern Hemisphere, and dispersal to the Northern Hemisphere has not been reported (*5*). By contrast, California sea lions are of the genus *Zalophus*, breed in coastal and offshore islands of California (USA) in the Northern Hemisphere, and disperse farther north after breeding season (*40*). Thus, the species are unlikely to have natural encounters.

We detected a nonhemotropic mycoplasma in blood and available frozen lung, spleen, and liver tissues from a Franciscana dolphin and frozen brain, lung, and spleen tissues from its calf, indicating systemic infection. Nonhemotropic mycoplasmas have already been detected in other marine mammals, including cetaceans, and were mainly associated with pneumonia and septic polyarthritis (*29*–*35*). Despite those detections, little information is available about the pathologic signature of these bacteria because the descriptions are usually linked with co-infections, like avian influenza virus or parasites, that could influence the observed lesions (*29*,*31*,*41*,*42*). In our study, both dolphins had mild to moderate granulocytic pneumonia with exfoliation of macrophages and pneumocytes in alveoli (Appendix Figure 2). Extensive pulmonary lesions, including pneumonia with granulocytes in the alveoli (*43*,*44*), have already been described, suggesting that the lesions observed in our study could be associated with mycoplasma infection. Furthermore, the brain of the calf tested positive for *Mycoplasma* spp. and demonstrated lesions compatible with neuronal suffering (e.g., neuronophagia). Detection of nonhemotropic mycoplasma in brain has been described in multiple mammals, including humans, seals, cattle, and sheep, and has been recorded mainly in nurslings (i.e., human infants and calves) (*23*), as observed in this investigation. In sheep, evidence of transmammary transmission and development of encephalitis in the infected lambs has been described (*23*). Despite isolation from the brains of harbor seals in an epizootic mortality episode in the North Sea 30 years ago, the role of mycoplasmas in central nervous system infections has not been clarified (*29*). Previous reports of nonhemotropic mycoplasmas in cetaceans were all in species distributed in the Northern Hemisphere, and detection was from lungs, nasopharynx, liver, preputium, and atlantooccipital joints (*31*,*33*,*34*).

We did not find statistically significantly higher hemoplasma occurrence in adults than in juveniles or calves, which differs from what we observed in Amazon (*Inia geoffrensis*) and Bolivian (*I. boliviensis*) river dolphins (*13*). Nevertheless, we detected hemoplasma in blood sampled over the years. Thus, endemicity of detected hemoplasmas in those populations cannot yet be determined. As we observed in Amazonian manatees (*Trichechus inunguis*) (*13*), all the West Indian manatees tested herein were PCR negative for mycoplasma. Manatees, unlike cetaceans and pinnipeds, are herbivorous and shed different endoparasites than the other 2 groups, which could explain the lack of mycoplasma-positive manatees (*45*). Nevertheless, most of the tested mammals had previously stranded as neonates or calves; therefore, they would have had little contact with adult manatees that could be a source of infection.

Some of the species selected for this study are threatened and experiencing decreasing population trends. The Franciscana dolphin and the West Indian manatee are currently classified as vulnerable by the International Union for Conservation of Nature’s Red List of Threatened Species (*7*). Certain hemoplasma species can cause disease and even death in immunosuppressed mammals, so those pathogens could potentially have conservation implications in aquatic mammals. That observation is especially crucial when considering that aquatic mammals are facing diverse anthropogenic and natural threats, such as aquatic pollution, climate change, and anthropization, which are capable of affecting their immune status and increasing disease susceptibility (*46*).

In conclusion, we detected *Mycoplasma* DNA in blood samples of marine cetaceans and pinnipeds in Brazil. Our findings indicate that at least 2 divergent species of hemotropic mycoplasma are circulating in cetaceans. In addition, we detected a nonhemotropic mycoplasma in Franciscana dolphins. The *Mycoplasma* sequences retrieved from pinnipeds were very similar to sequences previously described in California sea lions. Mycoplasmas were not detected in any of the tested sirenians. Our findings demonstrate a wider host range of hemotropic mycoplasma in cetaceans and pinnipeds and expand epitheliotropic mycoplasmas to cetaceans of the Southern Hemisphere, reinforcing the presence of those bacteria in aquatic mammals under natural conditions. The interspecies transmission, zoonotic potential and pathogenicity of all mycoplasmas detected should prompt additional serosurveys to elucidate the range and possible implications of *Mycoplasma* infections for marine mammals, especially endangered species.

Appendix Additional information on molecular detection and characterization of Mycoplasma spp. in marine mammals, Brazil.

## References

[1] Berta A, Sumich JL, Kovacs KM. Marine mammals: evolutionary biology, 2nd edition. London: Elsevier; 2005.

[2] Society for Marine Mammalogy. List of marine mammal species and subspecies, 2023 [cited 2023 Jun 20]. https://marinemammalscience.org/science-and-publications/list-marine-mammal-species-subspecies

[3] Moore SE. Marine mammals as ecosystem sentinels. J Mammal. 2008;89:534–40. 10.1644/07-MAMM-S-312R1.1

[4] Bossart GD. Marine mammals as sentinel species for oceans and human health. Vet Pathol. 2011;48:676–90. 10.1177/0300985810388525 21160025

[5] Frainer G, Heissler VL, Moreno IB. A wandering Weddell seal (*Leptonychotes weddellii*) at Trindade Island, Brazil: the extreme sighting of a circumpolar species. Polar Biol. 2018;41:579–82. 10.1007/s00300-017-2218-9

[6] Monteiro-Filho E, De Oliveira L, Monteiro K, Filla G, Quito L, Ferro De Godoy D; Cananéia Research Institute. Illustrated guide to marine mammals of Brazil [in Portuguese] [cited 2023 Apr 14]. https://ipecpesquisas.org.br/wp-content/uploads/2021/03/Guia-Ilustrado-2021-3_interativo_reduzido.pdf

[7] International Union for Conservation of Nature and Natural Resources. 2021 IUCN red list of threatened species [cited 2021 Jun 18]. https://www.iucnredlist.org

[8] Chico Mendes Institute for Biodiversity Conservation. National list of endangered species 2022 [in Portuguese] [cited 2023 Jun 26]. https://www.icmbio.gov.br/cepsul/images/stories/legislacao/Portaria/2020/P_mma_148_2022_altera_anexos_P_mma_443_444_445_2014_atualiza_especies_ameacadas_extincao

[9] Smith KF, Acevedo-Whitehouse K, Pedersen AB. The role of infectious diseases in biological conservation. Anim Conserv. 2009;12:1–12. 10.1111/j.1469-1795.2008.00228.x

[10] Groch KR, Santos-Neto EB, Díaz-Delgado J, Ikeda JMP, Carvalho RR, Oliveira RB, et al. Guiana dolphin unusual mortality event and link to cetacean morbillivirus, Brazil. Emerg Infect Dis. 2018;24:1349–54. 10.3201/eid2407.180139 29912687 PMC6038766

[11] Sacristán C, Esperón F, Ewbank AC, Costa-Silva S, Marigo J, Matushima ER, et al. Identification of novel gammaherpesviruses in a South American fur seal (*Arctocephalus australis*) with ulcerative skin lesions. J Wildl Dis. 2018;54:592–6. 10.7589/2017-09-224 29595382

[12] Sacristán C, Esperón F, Ewbank AC, Díaz-Delgado J, Ferreira-Machado E, Costa-Silva S, et al. Novel herpesviruses in riverine and marine cetaceans from South America. Acta Trop. 2019;190:220–7. 10.1016/j.actatropica.2018.11.021 30465743

[13] Duarte-Benvenuto A, Sacristán C, Ewbank AC, Sacristán I, Zamana-Ramblas R, Gravena W, et al. Hemotropic *Mycoplasma* spp. in aquatic mammals, Amazon Basin, Brazil. Emerg Infect Dis. 2022;28:2556–9. 10.3201/eid2812.220971 36418008 PMC9707567

[14] Ewbank AC, Duarte-Benvenuto A, Zamana-Ramblas R, Sacristán I, Costa-Silva S, Carvalho VL, et al. Herpesvirus and adenovirus surveillance in threatened wild West Indian (*Trichechus manatus*) and Amazonian manatees (*Trichechus inunguis*), Brazil. Acta Trop. 2023;237:106740. 10.1016/j.actatropica.2022.106740 36332674

[15] Brown DR, May M, Bradbury JM, Balish MF, Calcutt MJ, Glass JI, et al. Mycoplasma. In: Whitman WB, DeVos P, Dedysh S, Hedlund B, Kämpfer P, Rainey F, et al. editors. Bergey’s manual of systematics of archaea and bacteria. New York: Springer Science+Business Media; 2015. p. 1–78.

[16] Pitcher DG, Nicholas RAJ. Mycoplasma host specificity: fact or fiction? Vet J. 2005;170:300–6. 10.1016/j.tvjl.2004.08.011 16266844

[17] dos Santos AP, dos Santos RP, Biondo AW, Dora JM, Goldani LZ, de Oliveira ST, et al. Hemoplasma infection in HIV-positive patient, Brazil. Emerg Infect Dis. 2008;14:1922–4. 10.3201/eid1412.080964 19046522 PMC2634649

[18] Kumar A, Rahal A, Chakraborty S, Verma AK, Dhama K. Mycoplasma agalactiae, an etiological agent of contagious agalactia in small ruminants: a review. Vet Med Int. 2014;2014:286752. 10.1155/2014/286752 25097796 PMC4109668

[19] Millán J, Di Cataldo S, Volokhov DV, Becker DJ. Worldwide occurrence of haemoplasmas in wildlife: Insights into the patterns of infection, transmission, pathology and zoonotic potential. Transbound Emerg Dis. 2021;68:3236–56. 10.1111/tbed.13932 33210822

[20] Willi B, Boretti FS, Tasker S, Meli ML, Wengi N, Reusch CE, et al. From *Haemobartonella* to hemoplasma: molecular methods provide new insights. [b]. Vet Microbiol. 2007;125:197–209. 10.1016/j.vetmic.2007.06.027 17706380

[21] Razin S, Yogev D, Naot Y. Molecular biology and pathogenicity of mycoplasmas. Microbiol Mol Biol Rev. 1998;62:1094–156. 10.1128/MMBR.62.4.1094-1156.1998 9841667 PMC98941

[22] Hitti J, Garcia P, Totten P, Paul K, Astete S, Holmes KK. Correlates of cervical *Mycoplasma genitalium* and risk of preterm birth among Peruvian women. Sex Transm Dis. 2010;37:81–5. 10.1097/OLQ.0b013e3181bf5441 20051932 PMC4623580

[23] Rosales RS, Puleio R, Loria GR, Catania S, Nicholas RAJ. Mycoplasmas: Brain invaders? Res Vet Sci. 2017;113:56–61. 10.1016/j.rvsc.2017.09.006 28889017

[24] Sykes JE, Tasker S. Hemoplasma infections. In: Sykes J, editor. Canine and feline infectious diseases. St. Louis: Saunders (Elsevier): 2015. p. 390–8.

[25] Cabello J, Altet L, Napolitano C, Sastre N, Hidalgo E, Dávila JA, et al. Survey of infectious agents in the endangered Darwin’s fox (*Lycalopex fulvipes*): high prevalence and diversity of hemotrophic mycoplasmas. Vet Microbiol. 2013;167:448–54. 10.1016/j.vetmic.2013.09.034 24176254

[26] Di Cataldo S, Hidalgo-Hermoso E, Sacristán I, Cevidanes A, Napolitano C, Hernández CV, et al. Hemoplasmas are endemic and cause asymptomatic infection in the endangered Darwin’s fox (*Lycalopex fulvipes*). Appl Environ Microbiol. 2020;86:e00779–20. 10.1128/AEM.00779-20 32276983 PMC7267207

[27] Maggi RG, Compton SM, Trull CL, Mascarelli PE, Mozayeni BR, Breitschwerdt EB. Infection with hemotropic *Mycoplasma* species in patients with or without extensive arthropod or animal contact. J Clin Microbiol. 2013;51:3237–41. 10.1128/JCM.01125-13 23863574 PMC3811635

[28] Volokhov DV, Norris T, Rios C, Davidson MK, Messick JB, Gulland FM, et al. Novel hemotrophic mycoplasma identified in naturally infected California sea lions (*Zalophus californianus*). Vet Microbiol. 2011;149:262–8. 10.1016/j.vetmic.2010.10.026 21111543

[29] Giebel J, Meier J, Binder A, Flossdorf J, Poveda JB, Schmidt R, et al. *Mycoplasma phocarhinis* sp. nov. and *Mycoplasma phocacerebrale* sp. nov., two new species from harbor seals (*Phoca vitulina L.*). Int J Syst Bacteriol. 1991;41:39–44. 10.1099/00207713-41-1-39 1995034

[30] Ruhnke HL, Madoff S. *Mycoplasma phocidae* sp. nov., isolated from harbor seals *(Phoca vitulina L.*). Int J Syst Bacteriol. 1992;42:211–4. 10.1099/00207713-42-2-211 1581181

[31] Foster G, McAuliffe L, Dagleish MP, Barley J, Howie F, Nicholas RAJ, et al. Mycoplasma species isolated from harbor porpoises (*Phocoena phocoena*) and a Sowerby’s beaked whale (*Mesoplodon bidens*) stranded in Scottish waters. J Wildl Dis. 2011;47:206–11. 10.7589/0090-3558-47.1.206 21270010

[32] Lynch M, Taylor TK, Duignan PJ, Swingler J, Marenda M, Arnould JP, et al. Mycoplasmas in Australian fur seals (*Arctocephalus pusillus doriferus*): identification and association with abortion. J Vet Diagn Invest. 2011;23:1123–30. 10.1177/1040638711425699 22362792

[33] Díaz-Delgado J, Fernández A, Sierra E, Sacchini S, Andrada M, Vela AI, et al. Pathologic findings and causes of death of stranded cetaceans in the Canary Islands (2006-2012). PLoS One. 2018;13:e0204444. 10.1371/journal.pone.0204444 30289951 PMC6173391

[34] Marón CF, Kohl KD, Chirife A, Di Martino M, Fons MP, Navarro MA, et al. Symbiotic microbes and potential pathogens in the intestine of dead southern right whale (*Eubalaena australis*) calves. Anaerobe. 2019;57:107–14. 10.1016/j.anaerobe.2019.04.003 30959166

[35] White CP, Jewer DD. Seal finger: A case report and review of the literature. Can J Plast Surg. 2009;17:133–5. 10.1177/229255030901700415 21119845 PMC2827281

[36] Geraci JR, Lounsbury VJ. Marine mammals ashore: a field guide for strandings, 2nd ed. College Station (TX): Texas A&M University; 2005.

[37] Harasawa R, Orusa R, Giangaspero M. Molecular evidence for hemotropic *Mycoplasma* infection in a Japanese badger (*Meles meles anakuma*) and a raccoon dog (*Nyctereutes procyonoides viverrinus*). J Wildl Dis. 2014;50:412–5. 10.7589/2013-09-229 24484489

[38] Mongruel ACB, Spanhol VC, Valente JDM, Porto PP, Ogawa L, Otomura FH, et al. Survey of vector-borne and nematode parasites involved in the etiology of anemic syndrome in sheep from Southern Brazil. Rev Bras Parasitol Vet. 2020;29:e007320. 10.1590/s1984-29612020062 32935770

[39] Kumar S, Stecher G, Tamura K. MEGA7: Molecular Evolutionary Genetics Analysis version 7.0 for bigger datasets. Mol Biol Evol. 2016;33:1870–4. 10.1093/molbev/msw054 27004904 PMC8210823

[40] Carretta JV, Forney KA, Muto MM, Barlow J, Baker J, Hanson B, et al. Pacific marine mammal stock assessments, technical memorandum, NOAA-TM-NMFS-SWSC. Washington: National Oceanic and Atmospheric Administration; 2006.

[41] Haulena M, Gulland FM, Lawrence JA, Fauquier DA, Jang S, Aldridge B, et al. Lesions associated with a novel *Mycoplasma* sp. in California sea lions (*Zalophus californianus*) undergoing rehabilitation. J Wildl Dis. 2006;42:40–5. 10.7589/0090-3558-42.1.40 16699147

[42] Berhane Y, Joseph T, Lung O, Embury-Hyatt C, Xu W, Cottrell P, et al. Isolation and characterization of novel reassortant influenza A(H10N7) virus in a harbor seal, British Columbia, Canada. Emerg Infect Dis. 2022;28:1480–4. 10.3201/eid2807.212302 35731188 PMC9239883

[43] Bölske G, Engvall A, Renström LH, Wierup M. Experimental infections of goats with *Mycoplasma mycoides* subspecies mycoides, LC type. Res Vet Sci. 1989;46:247–52. 10.1016/S0034-5288(18)31153-6 2649950

[44] Dawood A, Algharib SA, Zhao G, Zhu T, Qi M, Delai K, et al. Mycoplasmas as host pantropic and specific pathogens: clinical implications, gene transfer, virulence factors, and future perspectives. Front Cell Infect Microbiol. 2022;12:855731. 10.3389/fcimb.2022.855731 35646746 PMC9137434

[45] Campbell HW, Irvine AB. Feeding ecology of the West Indian manatee *Trichechus manatus Linnaeus.* Aquaculture. 1977;12:249–51. 10.1016/0044-8486(77)90065-5

[46] Sanderson CE, Alexander KA. Unchartered waters: Climate change likely to intensify infectious disease outbreaks causing mass mortality events in marine mammals. Glob Change Biol. 2020;26:4284–301. 10.1111/gcb.15163 32558115

