# Supplementary material for: Molecular Detection and Characterization of Mycoplasma spp. in Marine Mammals, Brazil
Source: Emerg Infect Dis. 2023 Dec;29(12):2471–81. doi: 10.3201/eid2912.230903 (PMC10683811; doi:10.3201/eid2912.230903)
Supplement: Appendix — Additional information on molecular detection and characterization of Mycoplasma spp. in marine mammals, Brazil. [file 23-0903-Techapp-s1.pdf]

Article DOI: https://doi.org/10.3201/eid2912.230903

EID cannot ensure accessibility for supplementary materials supplied by authors. Readers who have difficulty accessing supplementary content should contact the authors for assistance.

Molecular Detection and Characterization of *Mycoplasma* spp. in Marine Mammals, Brazil

Appendix

Appendix Table. Epidemiologic and biologic data of marine mammals tested for *Mycoplasma* spp.

ID	Species	Family	Age class/sex	Stranded or bycatch	Sampling year	Habitat range	Latitude, longitude	Region	State
1	*Kogia breviceps*	Kogiidae	Adult/M	Stranded	2017	Off-shore	−4.757222, −37.279444	Northeast	Ceará
2	*Peponocephala electra*	Delphinidae	Juvenile/M	Stranded	2018	Off-shore	NR	Northeast	Ceará
3	*Peponocephala electra*	Delphinidae	Juvenile/M	Stranded	2018	Off-shore	NR	Northeast	Ceará
4	*Peponocephala electra*	Delphinidae	Juvenile/M	Stranded	2018	Off-shore	NR	Northeast	Ceará
5	*Peponocephala electra*	Delphinidae	Juvenile/M	Stranded	2018	Off-shore	NR	Northeast	Ceará
6	*Stenella coeruleoalba*	Delphinidae	Juvenile/F	Stranded	2018	Off-shore	NR	Northeast	Ceará
7	*Physeter macrocephalus*	Physeteridae	Calf/F	Stranded	2018	Off-shore	NR	Northeast	Ceará
8	*Peponocephala electra*	Delphinidae	Adult/M	Stranded	2018	Off-shore	NR	Northeast	Ceará
9	*Sotalia guianensis*	Delphinidae	Adult/F	Stranded	2018	Coastal	NR	Northeast	Ceará
10	*Sotalia guianensis*	Delphinidae	Calf/M	Stranded	2018	Coastal	NR	Northeast	Ceará
11	*Steno bredanensis*	Delphinidae	Adult/M	Stranded	2019	Mixed	NR	Northeast	Ceará
12	*Steno bredanensis*	Delphinidae	Adult/M	Stranded	2019	Mixed	NR	Northeast	Ceará
13	*Kogia sima*	Kogiidae	Adult/F	Stranded	2019	Off-shore	−4.631944, −37.491389	Northeast	Ceará
14	*Stenella coeruleoalba*	Delphinidae	Adult/F	Stranded	2019	Off-shore	−4.748056, −37.282778	Northeast	Ceará
15	*Kogia breviceps*	Kogiidae	Adult/M	Stranded	2020	Off-shore	−3.017778, −39.664444	Northeast	Ceará
16	*Kogia sima*	Kogiidae	Adult/M	Stranded	2020	Off-shore	−4.252778, −38.006944	Northeast	Ceará
17	*Feresa attenuata*	Delphinidae	Calf/M	Stranded	2020	Off-shore	NR	Northeast	Ceará
18	*Kogia sima*	Kogiidae	Calf/M	Stranded	2020	Off-shore	NR	Northeast	Ceará
19	*Stenella frontalis*	Delphinidae	Adult/F	Stranded	2020	Mixed	−2.848889, −40.603333	Northeast	Ceará
20	*Kogia sima*	Kogiidae	Juvenile/F	Stranded	2020	Off-shore	NR	Northeast	Ceará
21	*Feresa attenuata*	Delphinidae	Adult/F	Stranded	2020	Off-shore	NR	Northeast	Ceará
22	*Globicephala macrorhynchus*	Delphinidae	Juvenile/F	Stranded	2020	Off-shore	NR	Northeast	Ceará
23	*Globicephala macrorhynchus*	Delphinidae	Calf/F	Stranded	2020	Off-shore	NR	Northeast	Ceará
24	*Sotalia guianensis*	Delphinidae	Adult/F	Stranded	2021	Coastal	NR	Northeast	Ceará

ID	Species	Family	Age class/sex	Stranded or bycatch	Sampling year	Habitat range	Latitude, longitude	Region	State
25	*Sotalia guianensis*	Delphinidae	Calf/F	Stranded	2021	Coastal	NR	Northeast	Ceará
26	*Kogia breviceps*	Kogiidae	Adult/F	Stranded	2020	Off-shore	−24.8038, −47.6303	Southeast	São Paulo
27	*Sotalia guianensis*	Delphinidae	Juvenile/M	Stranded	2020	Coastal	NR	Southeast	São Paulo
28	*Delphinus delphis*	Delphinidae	Juvenile/F	Stranded	2021	Mixed	NR	Southeast	São Paulo
29	*Stenella frontalis*	Delphinidae	Adult/F	Stranded	2021	Mixed	−24.9797309, −47.8463685	Southeast	São Paulo
30	*Stenella clymene*	Delphinidae	Adult/M	Stranded	2011	Off-shore	NR	Northeast	Ceará
31	*Kogia breviceps*	Kogiidae	Calf/M	Stranded	2012	Off-shore	NR	Northeast	Ceará
32	*Peponocephala electra*	Delphinidae	Adult/F	Stranded	2011	Off-shore	NR	Northeast	Ceará
33	*Kogia sima*	Kogiidae	Adult/M	Stranded	2012	Off-shore	NR	Northeast	Ceará
34	*Feresa attenuata*	Delphinidae	Adult/M	Stranded	2015	Off-shore	−3.506111, −38.905278	Northeast	Ceará
35	*Sotalia guianensis*	Delphinidae	Calf/F	Stranded	2011	Coastal	NR	Northeast	Ceará
36	*Kogia sima*	Kogiidae	Adult/F	Stranded	2013	Off-shore	NR	Northeast	Ceará
37	*Peponocephala electra*	Delphinidae	Juvenile/M	Stranded	2011	Off-shore	NR	Northeast	Ceará
38	*Kogia sima*	Kogiidae	Adult/F	Stranded	2013	Off-shore	NR	Northeast	Ceará
39	*Kogia sima*	Kogiidae	Calf/M	Stranded	2013	Off-shore	NR	Northeast	Ceará
40	*Peponocephala electra*	Delphinidae	Calf/M	Stranded	2012	Off-shore	NR	Northeast	Ceará
41	*Grampus griseus*	Delphinidae	Adult/F	Stranded	2013	Off-shore	NR	Northeast	Ceará
42	*Stenella longirostris*	Delphinidae	Juvenile/M	Stranded	2015	Off-shore	NR	Northeast	Ceará
43	*Sotalia guianensis*	Delphinidae	Calf/F	Stranded	2015	Coastal	NR	Northeast	Ceará
44	*Kogia sima*	Kogiidae	Adult/M	Stranded	2015	Off-shore	NR	Northeast	Ceará
45	*Sotalia guianensis*	Delphinidae	Juvenile/F	Stranded	2018	Coastal	NR	Southeast	São Paulo
46	*Pontoporia blainvillei*	Pontoporiidae	Adult/F	Bycatch	2019	Coastal	NR	Southeast	São Paulo
47	*Pontoporia blainvillei*	Pontoporiidae	Adult/M	Stranded	2020	Coastal	NR	Southeast	São Paulo
48	*Pontoporia blainvillei*	Pontoporiidae	Juvenile/F	Bycatch	2019	Coastal	NR	Southeast	São Paulo
49	*Pontoporia blainvillei*	Pontoporiidae	Calf/F	Stranded	2019	Coastal	NR	Southeast	São Paulo
50	*Pontoporia blainvillei*	Pontoporiidae	Juvenile/M	Stranded	2020	Coastal	NR	Southeast	São Paulo
51	*Pontoporia blainvillei*	Pontoporiidae	Adult/M	Bycatch	2020	Coastal	NR	Southeast	São Paulo
52	*Sotalia guianensis*	Delphinidae	Juvenile/M	Stranded	2021	Coastal	NR	Southeast	São Paulo
53	*Pontoporia blainvillei*	Pontoporiidae	Calf/M	Stranded	2021	Coastal	NR	Southeast	São Paulo
54	*Pontoporia blainvillei*	Pontoporiidae	Adult/F	Bycatch	2021	Coastal	NR	Southeast	São Paulo
55	*Pontoporia blainvillei*	Pontoporiidae	Juvenile/M	Bycatch	2018	Coastal	NR	Southeast	São Paulo
56	*Pontoporia blainvillei*	Pontoporiidae	Adult/M	Bycatch	2020	Coastal	NR	Southeast	São Paulo
57	*Pontoporia blainvillei*	Pontoporiidae	Adult/F	Stranded	2020	Coastal	NR	Southeast	São Paulo
58	*Pontoporia blainvillei*	Pontoporiidae	Adult/F	Stranded	2019	Coastal	NR	Southeast	São Paulo
59	*Sotalia guianensis*	Delphinidae	Adult/M	Stranded	2021	Coastal	NR	Southeast	São Paulo
60	*Pontoporia blainvillei*	Pontoporiidae	Juvenile/F	Stranded	2019	Coastal	NR	Southeast	São Paulo
61	*Sotalia guianensis*	Delphinidae	Juvenile/M	Bycatch	2020	Coastal	NR	Southeast	São Paulo

ID	Species	Family	Age class/sex	Stranded or bycatch	Sampling year	Habitat range	Latitude, longitude	Region	State
62	*Delphinus delphis*	Delphinidae	Juvenile/M	Stranded	2021	Mixed	−24.030488, −46.4731254	Southeast	São Paulo
63	*Sotalia guianensis*	Delphinidae	Juvenile/F	Stranded	2021	Coastal	−24.3200518, −46.9893286	Southeast	São Paulo
64	*Pontoporia blainvillei*	Pontoporiidae	Juvenile/F	Bycatch	2021	Coastal	NR	Southeast	São Paulo
65	*Pontoporia blainvillei*	Pontoporiidae	Calf/F	Stranded	2021	Coastal	NR	Southeast	São Paulo
66	*Pontoporia blainvillei*	Pontoporiidae	Juvenile/F	Stranded	2021	Coastal	NR	Southeast	São Paulo
67	*Pontoporia blainvillei*	Pontoporiidae	Adult/F	Stranded	2021	Coastal	NR	Southeast	São Paulo
68	*Pontoporia blainvillei*	Pontoporiidae	Juvenile/M	Stranded	2021	Coastal	NR	Southeast	São Paulo
69	*Pontoporia blainvillei*	Pontoporiidae	Juvenile/F	Bycatch	2021	Coastal	NR	Southeast	São Paulo
70	*Pontoporia blainvillei*	Pontoporiidae	Calf/M	Stranded	2021	Coastal	NR	Southeast	São Paulo
71	*Pontoporia blainvillei*	Pontoporiidae	Adult/M	Stranded	2021	Coastal	NR	Southeast	São Paulo
72	*Pontoporia blainvillei*	Pontoporiidae	Calf/M	Stranded	2022	Coastal	NR	Southeast	São Paulo
73	*Pontoporia blainvillei*	Pontoporiidae	Calf/F	Stranded	2022	Coastal	NR	Southeast	São Paulo
74	*Pontoporia blainvillei*	Pontoporiidae	Juvenile/M	Bycatch	2022	Coastal	NR	Southeast	São Paulo
75	*Pontoporia blainvillei*	Pontoporiidae	Juvenile/M	Stranded	2018	Coastal	NR	Southeast	São Paulo
76	*Pontoporia blainvillei*	Pontoporiidae	Calf/M	Stranded	2018	Coastal	NR	Southeast	São Paulo
77	*Megaptera novaeangliae*	Balaenopteridae	Juvenile/M	Stranded	2018	Mixed	NR	Southeast	São Paulo
78	*Pontoporia blainvillei*	Pontoporiidae	Juvenile/M	Stranded	2018	Coastal	NR	Southeast	São Paulo
79	*Pontoporia blainvillei*	Pontoporiidae	Calf/M	Stranded	2019	Coastal	−24.0425891, −46.5055951	Southeast	São Paulo
80	*Pontoporia blainvillei*	Pontoporiidae	Calf/M	Stranded	2017	Coastal	NR	Southeast	São Paulo
81	*Steno bredanensis*	Delphinidae	Adult/M	Stranded	2019	Off-shore	−24.0149953, −46.4076126	Southeast	São Paulo
82	*Sotalia guianensis*	Delphinidae	Juvenile/M	Stranded	2018	Coastal	NR	Southeast	São Paulo
83	*Pontoporia blainvillei*	Pontoporiidae	Juvenile/F	Bycatch	2019	Coastal	NR	Southeast	São Paulo
84	*Pontoporia blainvillei*	Pontoporiidae	Juvenile/M	Bycatch	2018	Coastal	NR	Southeast	São Paulo
85	*Pontoporia blainvillei*	Pontoporiidae	Adult/F	Bycatch	2018	Coastal	−24.028428, −46.470938	Southeast	São Paulo
86	*Pontoporia blainvillei*	Pontoporiidae	Adult/F	Bycatch	2018	Coastal	NR	Southeast	São Paulo
87	*Pontoporia blainvillei*	Pontoporiidae	Adult/F	Bycatch	2019	Coastal	NR	Southeast	São Paulo
88	*Pontoporia blainvillei*	Pontoporiidae	Juvenile/F	Bycatch	2018	Coastal	NR	Southeast	São Paulo
89	*Stenella frontalis*	Delphinidae	Adult/F	Stranded	2019	Mixed	NR	Southeast	São Paulo
90	*Pontoporia blainvillei*	Pontoporiidae	Juvenile/F	Bycatch	2018	Coastal	NR	Southeast	São Paulo
91	*Pontoporia blainvillei*	Pontoporiidae	Adult/F	Bycatch	2019	Coastal	NR	Southeast	São Paulo
92	*Pontoporia blainvillei*	Pontoporiidae	Adult/M	Bycatch	2020	Coastal	NR	Southeast	São Paulo
93	*Pontoporia blainvillei*	Pontoporiidae	Adult/M	Bycatch	2020	Coastal	NR	Southeast	São Paulo
94	*Stenella frontalis*	Delphinidae	Adult/M	Stranded	2020	Mixed	NR	Southeast	São Paulo

ID	Species	Family	Age class/sex	Stranded or bycatch	Sampling year	Habitat range	Latitude, longitude	Region	State
95	*Orcinus orca*	Delphinidae	Juvenile/F	Stranded	2020	Mixed	−12.7002, −38.3253	Northeast	Bahia
96	*Feresa attenuata*	Delphinidae	Calf/M	Stranded	2020	Off-shore	NR	Northeast	Bahia
97	*Peponocephala electra*	Delphinidae	Juvenile/M	Stranded	2021	Off-shore	NR	Northeast	Bahia
98	*Stenella clymene*	Delphinidae	Juvenile/F	Stranded	2022	Off-shore	NR	Northeast	Bahia
99	*Stenella clymene*	Delphinidae	Juvenile/M	Stranded	2022	Off-shore	NR	Northeast	Bahia
100	*Sotalia guianensis*	Delphinidae	Juvenile/F	Stranded	2022	Coastal	NR	Northeast	Espiríto Santo
101	*Kogia sima*	Kogiidae	Adult/M	Stranded	2019	Off-shore	NR	Northeast	Bahia
102	*Megaptera novaeangliae*	Balaenopteridae	Calf/M	Stranded	2013	Mixed	NR	Northeast	Espiríto Santo
103	*Lagenodelphis hosei*	Delphinidae	Calf/M	Stranded	2021	Off-shore	NR	Southeast	São Paulo
104	*Lagenodelphis hosei*	Delphinidae	Calf/F	Stranded	2021	Off-shore	NR	Southeast	São Paulo
105	*Lagenodelphis hosei*	Delphinidae	Adult/F	Stranded	2021	Off-shore	NR	Southeast	São Paulo
106	*Lagenodelphis hosei*	Delphinidae	Adult/F	Stranded	2021	Off-shore	NR	Southeast	São Paulo
107	*Sotalia guianensis*	Delphinidae	Juvenile/M	Stranded	2021	Coastal	NR	Southeast	São Paulo
108	*Sotalia guianensis*	Delphinidae	Adult/M	Stranded	2021	Coastal	NR	Southeast	São Paulo
109	*Steno bredanensis*	Delphinidae	Adult/F	Stranded	2020	Off-shore	NR	Southeast	São Paulo
110	*Stenella frontalis*	Delphinidae	Calf/M	Stranded	2022	Mixed	NR	Southeast	São Paulo
111	*Lagenodelphis hosei*	Delphinidae	Juvenile/F	Stranded	2021	Off-shore	NR	Southeast	São Paulo
112	*Megaptera novaeangliae*	Balaenopteridae	Juvenile/M	Stranded	2021	Mixed	NR	Southeast	São Paulo
113	*Sotalia guianensis*	Delphinidae	Adult/F	Stranded	2021	Coastal	NR	Southeast	São Paulo
114	*Stenella frontalis*	Delphinidae	Adult/M	Stranded	2021	Mixed	NR	Southeast	São Paulo
115	*Pontoporia blainvillei*	Pontoporiidae	Juvenile/M	Stranded	2021	Coastal	NR	Southeast	São Paulo
116	*Sotalia guianensis*	Delphinidae	Adult/F	Stranded	2021	Coastal	NR	Southeast	São Paulo
117	*Megaptera novaeangliae*	Balaenopteridae	Juvenile/ND	Stranded	2016	Mixed	NR	Northeast	Bahia
118	*Megaptera novaeangliae*	Balaenopteridae	Calf/M	Stranded	2022	Mixed	NR	Northeast	Bahia
119	*Sotalia guianensis*	Delphinidae	Juvenile/M	Stranded	2022	Coastal	−18.73059, −39.74632	Northeast	Espiríto Santo
120	*Sotalia guianensis*	Delphinidae	Adult/F	Stranded	2022	Coastal	NR	Northeast	Espiríto Santo
121	*Sotalia guianensis*	Delphinidae	Adult/M	Stranded	2022	Coastal	NR	Northeast	Bahia
122	*Pontoporia blainvillei*	Pontoporiidae	Calf/F	Stranded	2022	Coastal	−18.41453, −39.69797	Northeast	Espiríto Santo
123	*Sotalia guianensis*	Delphinidae	Calf/F	Stranded	2022	Coastal	NR	Northeast	Espiríto Santo
124	*Pontoporia blainvillei*	Pontoporiidae	Juvenile/M	Stranded	2022	Coastal	NR	Northeast	Espiríto Santo
125	*Megaptera novaeangliae*	Balaenopteridae	Calf/M	Stranded	2013	Mixed	NR	Northeast	Bahia
126	*Sotalia guianensis*	Delphinidae	Juvenile/M	Stranded	2022	Coastal	−18.74685, −39.74704	Northeast	Espiríto Santo
127	*Peponocephala electra*	Delphinidae	Adult/M	Stranded	2022	Off-shore	NR	Northeast	Ceará
128	*Stenella frontalis*	Delphinidae	Adult/F	Stranded	2022	Mixed	NR	Northeast	Ceará
129	*Sotalia guianensis*	Delphinidae	Calf/F	Stranded	2022	Coastal	NR	Northeast	Ceará
130	*Stenella longirostris*	Delphinidae	Adult/M	Stranded	2022	Off-shore	NR	Northeast	Ceará

ID	Species	Family	Age class/sex	Stranded or bycatch	Sampling year	Habitat range	Latitude, longitude	Region	State
131	*Arctocephalus tropicalis*	Otariidae	Adult/M	Stranded	2020	Vagrant	−24.574229, −47.24243347	Southeast	São Paulo
132	*Arctocephalus tropicalis*	Otariidae	Adult/F	Stranded	2020	Vagrant	−24.702925, −47.46314	Southeast	São Paulo
133	*Arctocephalus australis*	Otariidae	Juvenile/M	Stranded	2020	Vagrant	NR	Southeast	São Paulo
134	*Arctocephalus australis*	Otariidae	Adult/F	Stranded	2020	Vagrant	NR	Southeast	São Paulo
135	*Arctocephalus australis*	Otariidae	Juvenile/M	Stranded	2020	Vagrant	NR	Southeast	São Paulo
136	*Arctocephalus gazela*	Otariidae	Juvenile/M	Stranded	2021	Vagrant	NR	Southeast	São Paulo
137	*Arctocephalus australis*	Otariidae	Juvenile/M	Stranded	2020	Vagrant	NR	Southeast	São Paulo
138	*Arctocephalus tropicalis*	Otariidae	Adult/F	Stranded	2020	Vagrant	NR	Southeast	São Paulo
139	*Arctocephalus tropicalis*	Otariidae	Adult/M	Stranded	2018	Vagrant	NR	Southeast	São Paulo
140	*Arctocephalus australis*	Otariidae	Juvenile/M	Stranded	2020	Vagrant	NR	Southeast	São Paulo
141	*Mirounga leonina*	Phocidae	Juvenile/F	Stranded	2022	Vagrant	NR	Northeast	Bahia
142	*Lobodon carcinophaga*	Phocidae	Juvenile/ND	Stranded	2021	Vagrant	NR	Southeast	São Paulo
143	*Mirounga leonina*	Phocidae	Adult/M	Stranded	2017	Vagrant	NR	Southeast	Espírito Santo
144	*Arctocephalus gazela*	Otariidae	Juvenile/M	Stranded	2021	Vagrant	−23.83144, −45.51249	Southeast	São Paulo
145	*Arctocephalus australis*	Otariidae	Juvenile/F	Stranded	2021	Vagrant	NR	Southeast	São Paulo
146	*Arctocephalus australis*	Otariidae	Juvenile/M	Stranded	2021	Vagrant	NR	Southeast	São Paulo
147	*Arctocephalus australis*	Otariidae	Juvenile/M	Stranded	2022	Vagrant	NR	Southeast	São Paulo
148	*Arctocephalus australis*	Otariidae	Juvenile/F	Stranded	2022	Vagrant	NR	Southeast	São Paulo
149	*Trichechus manatus*	Trichechidae	Adult/M	Stranded	2016	Coastal	NR	Northeast	Ceará
150	*Trichechus manatus*	Trichechidae	Neonate/F	Stranded	2016	Coastal	NR	Northeast	Ceará
151	*Trichechus manatus*	Trichechidae	Neonate/M	Stranded	2019	Coastal	NR	Northeast	Ceará
152	*Trichechus manatus*	Trichechidae	Neonate/F	Stranded	2016	Coastal	NR	Northeast	Ceará
153	*Trichechus manatus*	Trichechidae	Neonate/F	Stranded	2021	Coastal	NR	Northeast	Ceará
154	*Trichechus manatus*	Trichechidae	Neonate/M	Stranded	2002	Coastal	NR	Northeast	Ceará
155	*Trichechus manatus*	Trichechidae	Neonate/M	Stranded	2002	Coastal	NR	Northeast	Ceará
156	*Trichechus manatus*	Trichechidae	Neonate/F	Stranded	2008	Coastal	NR	Northeast	Ceará
157	*Trichechus manatus*	Trichechidae	Neonate/F	Stranded	2010	Coastal	NR	Northeast	Ceará
158	*Trichechus manatus*	Trichechidae	Neonate/M	Stranded	2011	Coastal	NR	Northeast	Ceará
159	*Trichechus manatus*	Trichechidae	Neonate/F	Stranded	2013	Coastal	NR	Northeast	Ceará
160	*Trichechus manatus*	Trichechidae	Neonate/M	Stranded	2013	Coastal	NR	Northeast	Ceará
161	*Trichechus manatus*	Trichechidae	Neonate/M	Stranded	2014	Coastal	NR	Northeast	Ceará
162	*Trichechus manatus*	Trichechidae	Neonate/M	Stranded	2014	Coastal	NR	Northeast	Ceará
163	*Trichechus manatus*	Trichechidae	ND/ND	Stranded	ND	Coastal	NR	Northeast	Ceará
164	*Trichechus manatus*	Trichechidae	Neonate/M	Stranded	2014	Coastal	NR	Northeast	Ceará

ID	Species	Family	Age class/sex	Stranded or bycatch	Sampling year	Habitat range	Latitude, longitude	Region	State
165	*Trichechus manatus*	Trichechidae	Neonate/F	Stranded	2015	Coastal	NR	Northeast	Ceará
166	*Trichechus manatus*	Trichechidae	Neonate/M	Stranded	2015	Coastal	NR	Northeast	Ceará
167	*Trichechus manatus*	Trichechidae	Neonate/M	Stranded	2016	Coastal	NR	Northeast	Ceará
168	*Trichechus manatus*	Trichechidae	Neonate/F	Stranded	2016	Coastal	NR	Northeast	Ceará
169	*Trichechus manatus*	Trichechidae	Neonate/F	Stranded	2017	Coastal	NR	Northeast	Ceará
170	*Trichechus manatus*	Trichechidae	Neonate/M	Stranded	2017	Coastal	NR	Northeast	Ceará
171	*Trichechus manatus*	Trichechidae	Calf/M	Stranded	2018	Coastal	NR	Northeast	Ceará
172	*Trichechus manatus*	Trichechidae	Adult/M	Stranded	2021	Coastal	NR	Northeast	Ceará
173	*Pontoporia blainvillei*	Pontoporiidae	Calf/F	Bycatch	2018	Coastal	NR	Southeast	São Paulo

*ND, not determined; NR, not recorded.

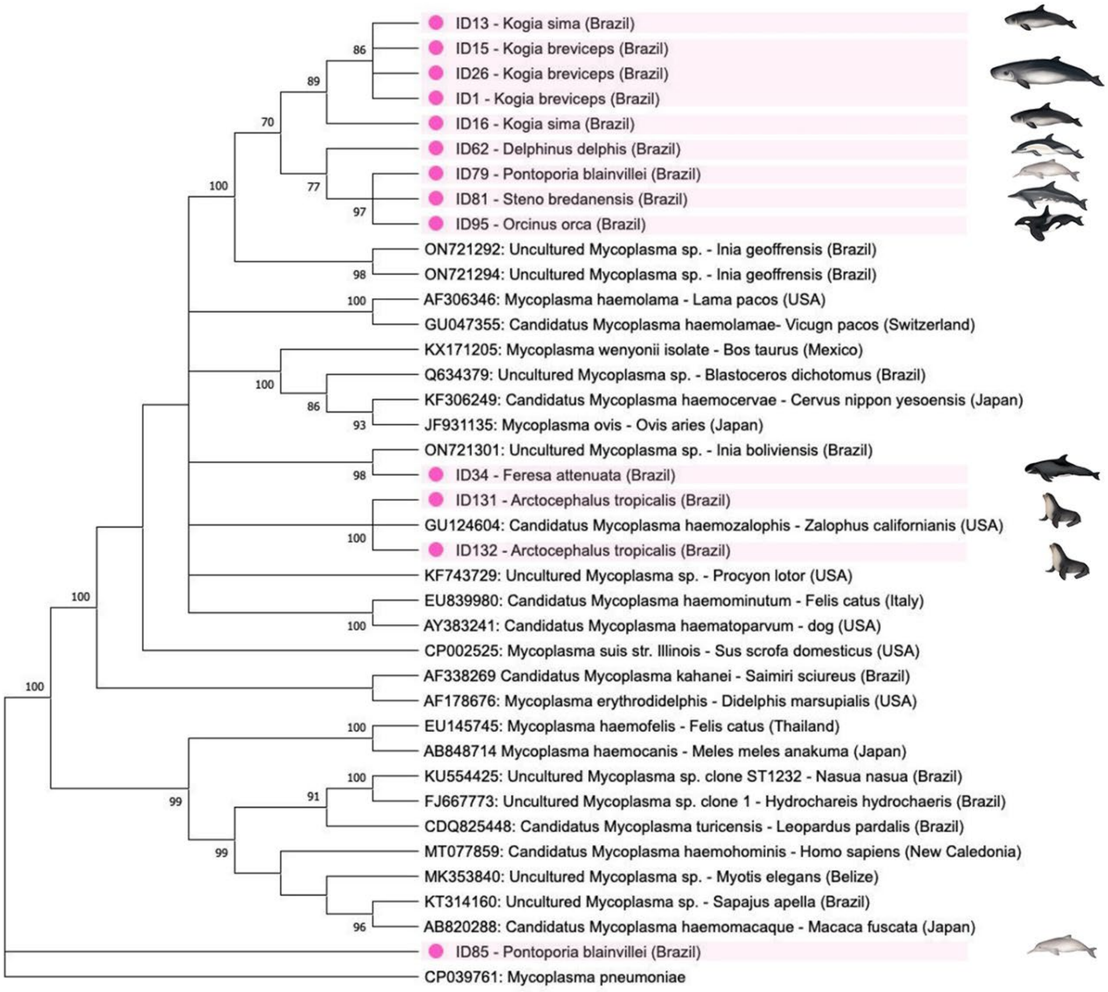

Appendix Figure 1. Maximum-likelihood phylogram from a study of molecular detection and characterization of *Mycoplasma* spp. in marine mammals, Brazil. Phylogram is based on the general time reversible plus inversions plus gamma distribution (GTR+I+G) evolutionary model of a 900-bp fragment of mycoplasma nucleotide sequences (pink dot) the obtained in this study and other hemotropic mycoplasma sequences retrieved from GenBank (https://www.ncbi.nlm.nih.gov/genbank). Reliability of the phylograms was tested by 1,000 replicate bootstrap analyses omitting values <70. Trees generate by using MEGA 7.0 (https://www.megasoftware.net). *Mycoplasma pneumoniae* was selected as an outgroup.

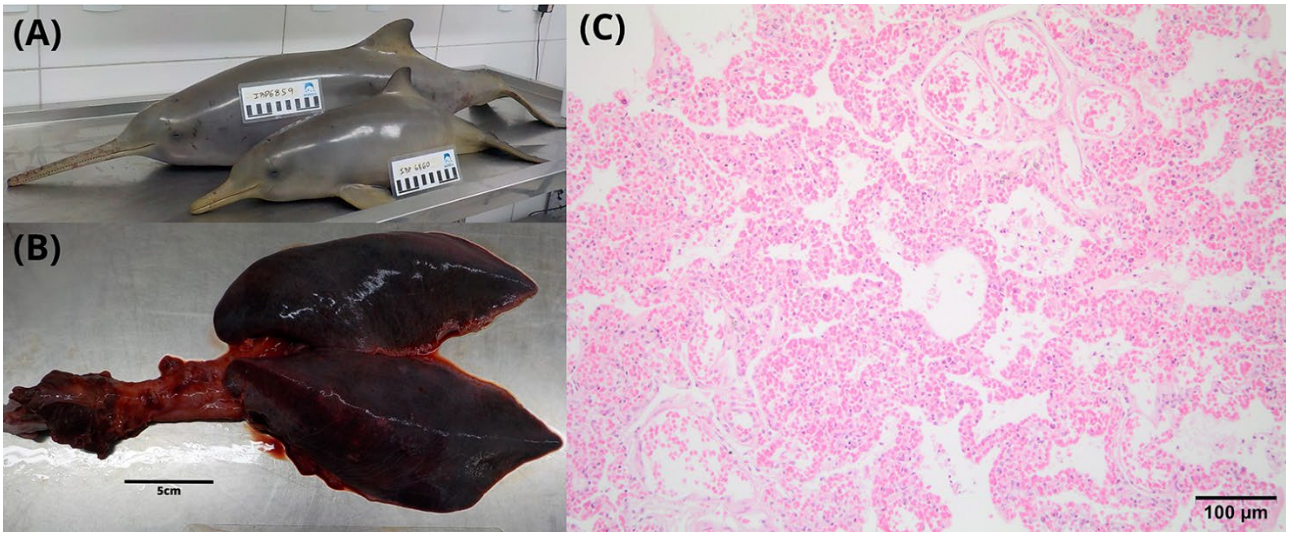

Appendix Figure 2. Gross and microscopic images of bycaught Franciscana dolphins (*Pontoporia blavinvillei)* positive sampled for a study of molecular detection and characterization of Mycoplasma spp. in marine mammals, Brazil. for epitheliotropic mycoplasma. A) Mother and calf. Note linear impressions in the rostrum of the adult female associated with net entanglement. B) Lungs of the adult female with congestion. C) Hematoxylin and eosin–stained histologic images of the lungs of the adult female. Note presence of intra-alveolar inflammatory exudate and cellular debris and congestion. Original magnification ×20.
